# Transient oculomotor paralysis after cerebral angiography: A case report

**DOI:** 10.1097/MD.0000000000026242

**Published:** 2021-06-04

**Authors:** Tao Qiu, Xiaoyan Dai, Qingping Gong, Rongmei Pu, Hua Xiao, Qiang Shi, Xiaoyong Deng, Ming Chen, Zhaoyun Guo

**Affiliations:** a Department of Neurology; b Equipment Management Department, Zigong First People's Hospital, Zigong, Sichuan, China.

**Keywords:** adverse reactions, contrast agent encephalitic, transient eye movement paralysis

## Abstract

**Rationale:**

A special case of transient oculomotor nerve palsy after cerebral angiography.

**Patient concerns:**

A 55-year-old man developed oculomotor nerve dysfunction after right radial artery puncture angiography.

**Diagnoses:**

Cerebral angiography-induced oculomotor nerve palsy.

**Interventions:**

According to the patient's disease state, intravenous drip of dexamethasone 10 mg/d.

**Outcomes:**

Magnetic resonance imaging (MRI) showed no abnormalities, and the patient recovered completely after 48 hours of hormone therapy.

**Lessons:**

Transient eye palsy caused by contrast agent encephalopathy is a clinically rare neurological dysfunction caused by adverse effects of contrast agents. Early prevention and correct treatment are critical.

## 1 Introduction

In recent years, with the increase of patients with carotid artery stenosis and intracranial aneurysms and the development of interventional techniques, cerebrovascular interventional therapy has become increasingly popular. Contrast agents in cerebrovascular interventional therapy can also cause contrast induced encephalopathy (CIE).^[1]^ This is a clinically rare complication, in which cortical blindness and epilepsy are common clinical manifestations of contrast-induced encephalopathy.^[2]^ However, there are few reports on eye movement paralysis, mainly after coronary angiography or treatment.^[3,4]^ Here, we report cases of transient oculomotor nerve palsy after cerebral angiography.

## 2 Case report

**Figure 1:**
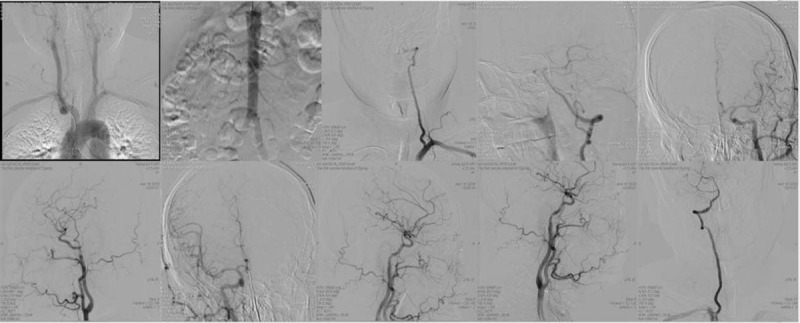
No obvious atherosclerotic plaque and vascular stenosis were found in the angiographic images of the patient.

The patient, a 55-year-old man, was admitted to hospital due to sudden dizziness for 10+ hours—the necessary blood pressure when admission was 130/86 mm Hg. The right radial artery puncture angiography was performed on the third day after admission (Fig. 1). The patient had a 9-year history of “type 2 diabetes” and took 1 metformin sustained-release tablets (qd). After admission, metformin sustained-release tablets were stopped and adjusted to acarbose 50 mg (oral medication before meals). Preoperative blood glucose was 10.9 mmol/L. With a history of penicillin allergy.

**Figure 2:**
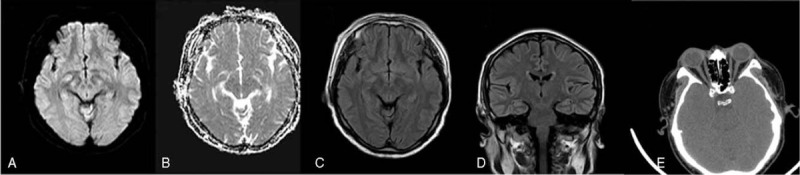
A–D. No obvious abnormality was found in emergency MRI; E. No obvious abnormality in orbital CT. CT = computed tomography, MRI = magnetic resonance imaging.

Ioversol was used in angiography with a total dose of 109 mL, in which 20 mL was used for renal arteriography, 89 mL was injected directly into the brain by the neck. The injection speed of aortic arch angiography was 12 mL/s, with the total amount of 20 mL and the pressure of 500 PSI. The injection speed of common carotid artery and subclavian artery angiography was 4 mL/s, with the total amount of 8 mL and the pressure of 200 PSI. During angiography, the blood pressure was 134/84 to 150/96 mm Hg. The diplopia occurred 7 hours after the operation. Physical examination: diplopia is found horizontally up, down, left, right, top, bottom right, left, right. Especially in the vertical direction, both pupils become larger and round. Sensitive to light reflection, pupil diameter is 3 mm, and there are no other positive signs. Blood glucose was 7.1 mmol/L. The oculomotor nerve (Cranial Nerve III) innervates the movement of the eye muscles and participates in the adjustment of reflex and pupil reflection to light. No obvious abnormalities were found in emergency magnetic resonance imaging (MRI) (Fig. 2A–D), so we believe that the patient's oculomotor nerve (Cranial Nerve III) has a transient paralysis. The diplopia was improved on the second day after an intravenous drip of dexamethasone 10 mg. Physical examination: there was diplopia in the lower, upper left, lower left, and left horizontal directions. Dexamethasone 10 mg was given intravenously again. On the third day after the operation, diplopia was relieved entirely and no abnormality was found in physical examination. Fundus photography: no bleeding or exudation was found. No defect was found in orbital computed tomography (CT) (Fig. 2E).

Clinically, for patients with cranial nerve dysfunction, various possible diseases of the brain should be considered (infection, vasculitis, subarachnoid hemorrhage, cerebral venous thrombosis, cerebral infarction, hypertensive emergency, reversible brain vasoconstriction syndrome, etc). The clinical presentation and brain imaging of this patient do not support the diagnosis of infection, vasculitis, subarachnoid hemorrhage, cerebral venous thrombosis, or cerebral infarction. Patients with non-acute hypertension also ruled out hypertensive emergencies. Reversible cerebral vasoconstriction syndrome, because he did not have recurrent thunderous headaches, and lack of typical risk factors were also excluded. Since the onset of the patient's brain is after the injection of contrast medium, the oculomotor nerve dysfunction caused by contrast medium-induced encephalopathy is strongly suspected.

**Figure 3:**
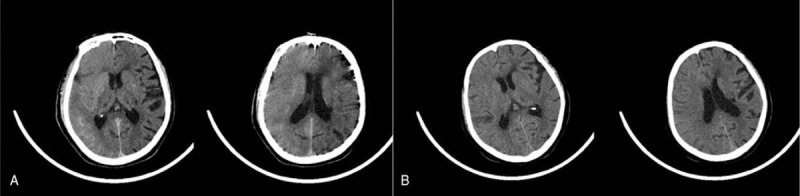
A. CT showed obvious swelling in the right hemisphere with high-density images, which was like subarachnoid hemorrhage. B. CT on the second day showed that the edema in the right hemisphere was significantly improved and the high-density image disappeared. CT = computed tomography.

After performing a cerebral angiography on a 74-year-old woman, we found that the patient had CIE. The patient had a right anterior communicating artery aneurysm and delirium in her previous medical history, and her left half of the brain was functioning normally. Emergency CT showed obvious edema in the right hemisphere, high-density imaging. An urgent review of cerebral angiography showed that the blood vessels were unobstructed and the aneurysm was not ruptured. The patient's symptoms improved significantly the next day, and high-density CT cerebral edema was significantly improved. On the 19th day after the onset of the disease, the patient was completely cured and discharged. The results of the 2 CTs are shown in Fig. 3.

## 3 Discussion

In angiography, contrast-induced neurotoxicity (CIN) is a very rare complication. Due to its low incidence, there are no specific statistics. However, some studies have reported the incidence of CIN: Spina et al^[3]^ found in a retrospective study of 6000 patients undergoing coronary angiography that the incidence rate was 0.15%. Data from articles published by Lantos^[5]^ in 1989, the estimated data is 1% to 2%. However, according to the author's >3000 cerebral angiography experiences. There are a total of 2 patients whose incidence should be similar to coronary angiography, which is much lower than the estimated data.

The clinical manifestations of CIN include encephalopathy, epilepsy, cortical blindness, focal neurological deficits, and cerebellar dysfunction.^[3]^ Fischer-Williams et al^[6]^ conducted the first clinical description of transient cortical blindness after X-ray irradiation in 1970. A retrospective study by Spina et al^[7]^ and Potsi et al^[8]^ found that cortical blindness is the most common manifestation, accounting for 52% (27/52). After performing a cerebral angiography on a 74-year-old woman, we found that the patient had CIE. The patient had a right anterior communicating artery aneurysm and delirium in her previous medical history, and her left half was not successful. Emergency CT showed obvious edema in the right hemisphere, high-density imaging. An urgent review of cerebral angiography showed that the blood vessels were unobstructed and the aneurysm was not ruptured. The patient's symptoms improved significantly the next day, and high-density CT cerebral edema was significantly improved. On the 19th day after the onset of the disease, the patient was completely cured and discharged. The results of the 2 CTs are shown in Fig. 3.

The cases of cranial nerve involvement in CIN are very rare. In 1989, Lantos^[5]^ reported that a 51-year-old man developed eye movement paralysis after carotid angiography. In 2010, Guimaraens et al^[9]^ reported that after a clavicle and vertebral arteriography, a 49-year-old woman and a 71-year-old man were involved in the cranial nerves under the clavicle and after aortic angiography. More cases were reported after coronary angiography or treatment. In 1990, Drummond and Wuebbolt^[10]^ reported that a 61-year-old woman developed bilateral ophthalmoplegia after coronary angiography. In 2013, Vasavada et al^[11]^ described a case of transient unilateral partial oculomotor nerve palsy after angioplasty. Eggenberger et al^[12]^ analyzed 110 patients who were diagnosed with internuclear ophthalmoplegia (INO) in 2 ophthalmic tissues during the observation period. Among them, 5 patients (4.5%) had relatively isolated INO during the perioperative period of intravascular surgery. Caillé et al^[13]^ and Yu and Dangas^[14]^ also reported a history of ophthalmoplegia after coronary angiography. In the above case studies of CIN cranial nerve involvement, cranial nerve palsy is usually short-lived, and most of them can recover on their own within 1 to 2 days. This recovery rate is like the results of this case analysis.

At present, the mechanism of CIN is unclear. Possible mechanisms at present: the destruction of the blood-brain barrier is related to osmotic pressure, ionicity, injection pressure, and dose of contrast medium, as well as other physical and chemical factors. It is generally believed that hypertonic contrast agents are more likely to break the blood-brain barrier. The high osmotic pressure allows the contrast agent to enter the brain cells through osmotic contraction, thereby opening the tight connection between the cerebral vascular endothelial cells. Some studies have shown that the incidence of CIN is between 0.3% and 1.0%, but when hypertonic iodine contrast agent is used, the incidence of CIN can reach 4%.^[15]^ In fact, osmotic pressure and ionicity are not necessary to destroy the blood-brain barrier, because encephalopathy can also occur in the case of isotonic non-ionic contrast agent iodixanol.^[16,17]^ In addition, physical factors such as increased intraluminal pressure caused by contrast injection may be one of the reasons for destroying the blood-brain barrier. Uncontrolled systemic blood pressure rises beyond the ability of cerebrovascular self-regulation. Elevated intraluminal pressure may lead to increased vascular wall tension, tight junction separation, rupture of the blood-brain barrier, and local exudation of fluids and contrast media.^[7]^

When it is suspected that CIE requires immediate CT scan of the brain to rule out ischemic and hemorrhagic stroke (including subarachnoid hemorrhage). The CT manifestations of CIE range from cortical and subcortical enhancement to focal high-density lesions, groove enhancement, cerebral edema, and subarachnoid space enhancement, like subarachnoid hemorrhage.^[18,19]^ Measuring CT value (HU) helps to distinguish different subarachnoid hemorrhages in CIE. Blood is usually 30 to 45 HU, and contrast agent is usually 80 to 160 HU.^[20]^ Pagani-Estévez et al^[21]^ uses a 3-substance decomposition algorithm based on brain parenchyma, hemorrhage, and iodine, and uses dual-energy iodine subtraction CT to analyze low-energy and high-energy images. CIE can be quickly diagnosed by virtual noncontrast and overlay images containing only iodine. In addition, the apparent diffusion coefficient (ADC) obtained from MRI is a method to quantitatively measure the diffusion of water molecules in tissues. And this is a reliable imaging method to distinguish contrast agent exudation from cerebral ischemia.^[2,22]^ In acute ischemic stroke with cytotoxic edema, the reduction of water diffusion in the infarcted tissue leads to a reduction in ADC. But in CIE, the ADC is normal. CIN is not always accompanied by typical radiological signs.^[1]^ For example, in this case report, there is no classic radiological manifestation.

Currently, there is no clear prevention and treatment method for CIE. Some researchers suggest that intravenous infusion and intra-arterial saline should be fully used during angiography. Intravenous steroids and mannitol may help control cerebral edema.^[2]^ However, some researchers also believe that these drugs have not shown to improve the prognosis.^[16]^ The prognosis of CIE is usually considered to be good and recover quickly, usually appearing and improving within 48 to 72 hours. However, some cases of CIE recover slowly. For example, the second case mentioned in our discussion section takes 19 days to recover, which may be related to re-exposure to contrast agent. In addition, some published cases show persistent neurological deficits.^[23]^ Therefore, we report here a special case of transient palsy caused by cerebral angiography. Hope to promote the understanding of the occurrence and development of CIE and improve the safety and reliability of cerebral angiography.

## 4 Conclusion

Oculomotor paralysis caused by cerebral angiography contrast medium is rarely reported and is a rare complication. It is a special manifestation of CIN. At present, its pathogenesis is unclear, so as its methods of treatment and prevention. But fortunately, most of the patients have a good prognosis. For interventional neurosurgeons, it is important to recognize the existence of this complication and avoid giving wrong treatment to patient.

## Author contributions

**Conceptualization:** Tao Qiu.

**Data curation:** Tao Qiu, Xiaoyan Dai, Qingping Gong, Qiang Shi, Xiaoyong Deng.

**Formal analysis:** Xiaoyan Dai.

**Investigation:** Rongmei Pu, Ming Chen.

**Project administration:** Hua Xiao.

**Writing – original draft:** Tao Qiu.

**Writing – review & editing:** Tao Qiu, Zhaoyun Guo.
